# Postoperative pain after cholecystectomy: Conventional laparoscopy versus single-incision laparoscopic surgery

**DOI:** 10.4103/0972-9941.72370

**Published:** 2011

**Authors:** A Prasad, K A Mukherjee, S Kaul, M Kaur

**Affiliations:** Department of Minimal Access Surgery, Indraprastha Apollo Hospital, New Delhi, India

**Keywords:** Laparoscopic cholecystectomy, single-incision laparoscopic surgery

## Abstract

**BACKGROUND:**

This study was undertaken to compare the postoperative pain after cholecystectomy done by single-incision laparoscopic surgery (SILS) versus conventional four-port laparoscopy [conventional laparoscopic surgery (CLS)]. SILS is a feasible and a promising method for cholecystectomy. It is possible to do this procedure without the use of special equipments. While there are cosmetic advantages to SILS, it is not clear whether or not the pain is also reduced.

**METHODS:**

Patients undergoing cholecystectomy for symptomatic gallstones were offered the choice of the two methods and the first 100 consecutive patients from each group were included in this observational study. Only conventional instruments were used to keep the cost of surgery comparable. Pain scores were checked 8 hours after the surgery using visual analogue score. Student’s t test was done to check the statistical significance.

**RESULTS:**

We observed no significant difference in the pain score between the CLS and SILS (2.78 versus 2.62). The operative time (OT) was significantly lower in the CLS group (28 versus 67 minutes). Comparing the OTs of the first 50 patients undergoing SILS with the second 50 patients showed a significantly lower OT (79 versus 54 minutes). We also compared the pain score between these three groups. The second half of SILS group had a significantly lower pain score compared to the first half (2.58 versus 2.84). This group also had a lower pain score compared to conventional laparoscopy group but the difference was not statistically significant (2.58 versus 2.78).

**CONCLUSION:**

Although there was no significant difference in the overall postoperative pain as OT decreases with surgeon’s experience in single-incision laparoscopic cholecystectomy, postoperative pain at 8 hours appears to favour this method over conventional laparoscopic cholecystectomy.

## INTRODUCTION

Laparoscopic cholecystectomy has replaced open cholecystectomy as the gold standard surgical procedure for majority of patients with gallstone disease.[1] Conventional laparoscopic cholecystectomy is done using four ports. With an effort to minimise the number of ports, single-incision laparoscopic surgery (SILS) has come into practice.[2] SILS is a rapidly evolving method that is complementing traditional laparoscopy in selected fields and patients.[3,4] It has also been suggested as a bridge between traditional laparoscopy and natural orifice transluminal endoscopic surgery (NOTES).[5]

SILS utilises three ports through a single skin incision at the umbilicus.[6] It is being considered as no-scar surgery because the incision is placed within the umbilical scar that is not visible.[7,8] SILS has also shown to have reduced postoperative pain as compared to four-port cholecystectomy in a recent randomised study, although the sample size was small.[9] Many special instruments[6] and ports[10,11] are available now for SILS. Technical modifications like puppeteering of the gallbladder with a suture have also been described.[12] We performed SILS cholecystectomy using only conventional laparoscopic instruments. The study compared the postoperative pain scores in patients undergoing SILS and conventional laparoscopic cholecystectomy.

## METHODS

The study was done at Indraprastha Apollo Hospital, New Delhi, India, from 1 September 2009 to 30 May 2010. We offer the options of conventional laparoscopic and single-incision laparoscopic cholecystectomy to patients undergoing cholecystectomy for symptomatic gallstones and the patients were allowed to choose the procedure. During this period, the first 100 consecutive patients from each group were included in this observational study. All patients with symptomatic cholelithiasis, who were fit for general anaesthesia, were included in the study and those with acute cholecystitis, abnormal liver function tests, those found to have a contracted or thickened gallbladder on the ultrasound examination and those with a suspicion of gallbladder malignancy were excluded.

Only conventional instruments were used in both the groups to keep the cost of surgery comparable. No special ports, roticulating instruments or flexible telescopes were used.

### Technique

A standard four-port cholecystectomy was done in the conventional laparoscopic surgery (CLS) group with two 10-mm ports in umbilical and epigastric locations along with two 5-mm ports in the right subcostal and lumbar regions.

**Figure 1:**
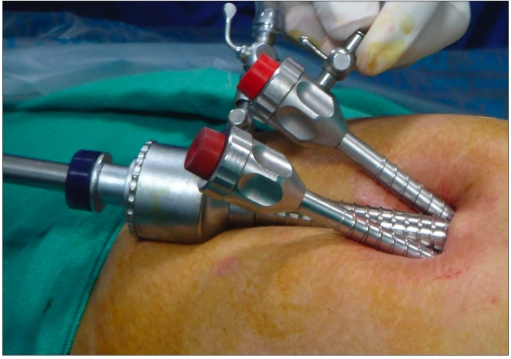
These ports were arranged in an inverted triangle

**Figure 2:**
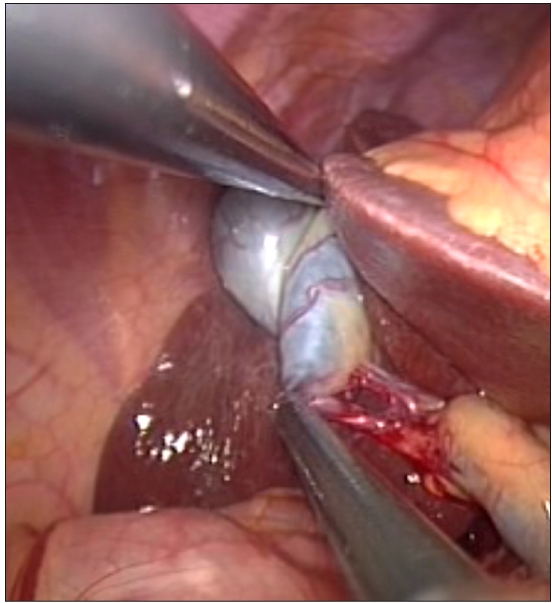
Dissection of the Calot’s triangle during SILS

**Figure 3:**
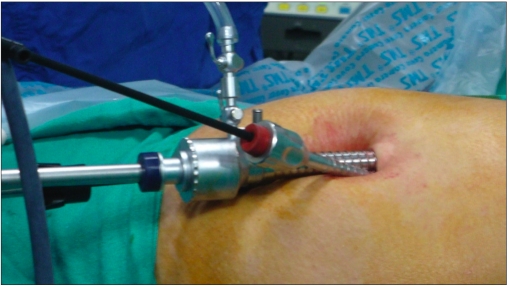
Chopstick method of crossing of instruments

In the SILS group, a 2-cm transverse incision was made at the level of the umbilicus. The upper skin flap was raised for a distance of 1 cm. After initial insufflation with Veress needle, a 10-mm port was inserted at the incision line and the two 5-mm ports were placed 0.5 cm inferiorly and laterally on either side through the same skin incision [Figure 1]. A grasper introduced through the right lateral port was used for fundal traction. The dissector introduced through the left lateral port was used to dissect the fine Calot’s triangle [Figure 2]. The instrument port and the telescope port were crossed by a chopstick method [Figure 3] to avoid “sword fighting” and clashing of instruments in the abdomen. The procedure was started with a 10-mm laparoscope which was later replaced by a 5-mm laparoscope from the left lateral port to allow use of a 10-mm clip applicator from the central port. After dissection of the gallbladder from the liver bed and achieving haemostasis, the gallbladder was delivered from the central port site. Fascial defects were closed individually and skin was apposed. All port sites were infiltrated with 0.5% Bupivacaine (Astra Zeneca, Mumbai, India). All patients received an intraoperative dose of 75 mg Diclofenac and the next dose was scheduled 12 hours later. The pain scores were checked 8 hours after surgery using a visual analogue score by a member of the team unaware of the type of procedure. Student’s t test was used for assessing the statistical significance.

## RESULTS

Two hundred patients undergoing cholecystectomy were included in the study and they were divided into two groups having 100 patients in each group. Group A comprised patients undergoing CLS and group B comprised those undergoing SILS. The groups were well matched for age and sex distribution as well as the body mass index (BMI) [Table 1].

**Table 1:** Demographic data of patients undergoing conventional and single-incision laparoscopy for cholecystectomy

	CLS (n = 100)	SILS (n = 100)	P†
Age* (years)	37.5	38.1	0.436
Sex ratio (M:F)	42:58	44:56	0.887‡
BMI*	27.3	27.7	0.497

* Values are mean; CLS, conventional laparoscopic surgery; SILS, singleincision laparoscopic surgery; BMI, body mass index

† *t*Test unless indicated otherwise

‡ 2 test

There were no conversions from SILS to CLS or conversion to open surgery. There were no intraoperative or postoperative complications.

The pain scores in both the groups were tabulated [Table 2]. The mean pain score was lower in the SILS group (2.62) as compared to the CLS group (2.78) but the difference did not reach statistical significance. The operative time (OT) was found to be significantly higher in the SILS group as compared to the CLS group [Table 3]. The mean OT in the CLS group was 28.08 ± 1.35 minutes while in the SILS group it was 66.76 ± 5.78 minutes. The mean OT in the first 50 cases of SILS (79.2 minutes) was significantly higher than that in the second 50 cases (54.32 minutes).

**Table 2:** Mean postoperative pain scores in the two groups of patients

	Number of patients	VAS score*	*P* value† (t test)	Statistical significance
Group A (CLS)	100	2.78	0.176	No
Group B (SILS)	100	2.62		
First half (SILS)	50	2.84	0.026	Yes
Second half (SILS)	50	2.58		
Group A (CLS)	100	2.78	0.051	No
Second half (SILS)	50	2.58		

* Values are mean; CLS, conventional laparoscopic surgery; SILS, singleincision laparoscopic surgery; VAS, visual analogue scale

† *t*Test

**Table 3:** Mean operative time in different groups of patients

	Number of patients	Operative time*	*P* value†	Statisticaiiy significant
Group A (CLS)	100	28.08	<0.05	Yes
Group B (SILS)	100	66.76		
First half SILS	50	79.2	<0.05	Yes
Second half SILS	50	54.32		
Group A (CLS)	100	28.08	<0.05	Yes
Second half SILS	50	54.32		

* Values are mean; CLS, conventional laparoscopic surgery; SILS, singleincision laparoscopic surgery

† *t*Test

On comparing the pain scores of the first 50 cases of SILS with the second 50 cases and the CLS group, we found that the second half of the SILS group had a significantly lower mean pain score (2.58) as compared to the first half (2.84). This group also had a lower pain score compared to the conventional laparoscopy group (2.78). Once again, the differences were not statistically significant.

## DISCUSSION

SILS is not a new concept and was described as early as 1992 by Pelosi *et al*.[2] who performed a single-puncture laparoscopic appendectomy. First experiences with SILS cholecystectomy were reported by Navarra *et al*. in 1997[3] and with a different approach by Piskun and Rajpal in 1999.[4]

In recent years, SILS has been seen as a bridge between NOTES and traditional laparoscopic surgery.[5] NOTES is a technically challenging procedure and current instruments need to be further improved.[13] SILS, on the other hand, allows the surgeon the freedom of using the existing laparoscopic instruments. The cited advantages of reducing the number of ports include better cosmesis, along with lowered wound complications, reduced morbidity of bleeding, incisional hernia and organ damage. Benefits of SILS in terms of reduced postoperative pain have not been confirmed.[14]

As most of the available special access devices and roticulating instruments are disposable, they add significantly to the cost of SILS. We used only traditional laparoscopic instruments and ports. We adopted indigenous methods such as applying adhesive dressings and gauze soaked with ointment around the cannulas to prevent air leak. The real challenge of SILS is to avoid conflict between the operative instruments and the camera, to maintain the pneumoperitoneum and reduce operative stress. As a result of the limited space with using only a single-incision, it is difficult for both the surgeon and the assistant to work in the area.[15] We have developed a chopstick method to minimise instrument and telescope clash during the procedure.

In our study, the postoperative pain was less in SILS group as compared to the CLS group, but not significantly so. The OTs, as expected, were higher in SILS group and this is in keeping with some of the recently published series on SILS cholecystectomy.[8,16] In the patients from the first half of SILS group, the OTs were longer than those in the patients from the second half. This can be explained on the basis of a “learning curve” effect. We found the postoperative pain to be significantly lower in the patients from the second half of the SILS group as compared to the first half patients. Also, this group had a lower pain score as compared to the CLS group. This difference, however, did not reach statistical significance. It is likely that the postoperative pain is multifactorial in its origin and OT may have some bearing on it. A study with a larger number of patients would be required to test whether with increasing experience and reduction in OT, the postoperative pain reduces significantly.

SILS for cholecystectomy appears to be a feasible and promising method for the treatment of symptomatic cholelithiasis.[17] This surgery can be performed safely with traditional reusable laparoscopic instruments.[18] Clashing of the instrument can be reduced by using the chopstick method described above.[19]

Although our study did not show a significant difference in the postoperative pain after SILS as compared to conventional laparoscopy, we feel that reduction in the OT with gained experience may reduce the postoperative pain. The drawback of our study that this is not a randmised trial and this can be overcome in the future by conducting a randomised trial with a larger number of patients. This may answer the question as to whether or not SILS carries a significant advantage in terms of postoperative pain over conventional laparoscopy.

## References

[1] Johnson CD (2001). ABC of the upper gastrointestinal tract Upper abdominal pain: Gall bladder. Br Med J.

[2] Pelosi MA, Pelosi MA (1992). Laparoscopic appendectomy using a single umbilical puncture (minilaparoscopy). J Reprod Med.

[3] Navarra G, Pozza E, Occhionorelli S, Carcoforo P, Donini I (1997). One-wound laparoscopic cholecystectomy. Br J Surg.

[4] Piskun G, Rajpal S (1999). Transumbilical laparoscopic cholecystectomy utilizes no incisions outside the umbilicus. J Laparoendosc Adv Surg Tech.

[5] Bresadola F, Pasqualucci A, Donini A, Chiarandini P, Anania G, Terrosu G (1999). Elective transumbilical compared with standard laparoscopic cholecystectomy. Eur J Surg.

[6] Tacchino R, Greco F, Matera D (2009). Single-incision laparoscopic cholecystectomy: Surgery without a visible scar. Surg Endosc.

[7] Cuesta MA, Berends F, Veenhof AA (2008). The “invisible cholecystectomy”: A transumbilical laparoscopic operation without a scar. Surg Endosc.

[8] Hong TH, You YK, Lee KH (2009). Transumbilical single-port laparoscopic cholecystectomy : Scarless cholecystectomy. Surg Endosc.

[9] Tsimoyiannis EC, Tsimogiannis KE, Pappas-Gogos G, Farantos C, Benetatos N, Mavridou P (2010). Different pain scores in single transumbilical incision laparoscopic cholecystectomy versus classic laparoscopic cholecystectomy: A randomized controlled trial. Surg Endosc.

[10] Romanelli JR, Mark L, Omotosho PA (2008). Single port laparoscopic cholecystectomy with the TriPort system: A case report. Surg Innov.

[11] Merchant AM, Cook MW, White BC, Davis SS, Sweeney JF, Lin E (2009). Transumbilical Gelport access technique for performing single-incision laparoscopic surgery (SILS). J Gastrointest Surg.

[12] Chow A, Purkayastha S, Aziz O, Paraskeva P (2010). Single-incision laparoscopic surgery for cholecystectomy: An evolving technique. Surg Endosc.

[13] Marescaux J, Dallemagne B, Perretta S, Wattiez A, Mutter D, Coumaros D (2007). Surgery without scars: Report of transluminal cholecystectomy in a human being. Arch Surg.

[14] Lee PC, Lo C, Lai PS, Chang JJ, Huang SJ, Lin MT (2010). Randomized clinical trial of single-incision laparoscopic cholecystectomy versus minilaparoscopic cholecystectomy. Br J Surg.

[15] Ishikawa N, Arano Y, Shimizu S, Morishita M, Kawaguchi M, Matsunoki A (2009). Single-incision laparoscopic surgery (SILS) using cross hand technique. Minim Invasive Ther Allied Technol.

[16] Kuon Lee S, You YK, Park JH, Kim HJ, Lee KK, Kim DG (2009). Single-port transumbilical laparoscopic cholecystectomy: A preliminary study in 37 patients with gallbladder disease. J Laparoendosc Adv Surg Tech A.

[17] Ersin S, Firat O, Sozbilen M (2010). Single-incision laparoscopic cholecystectomy: Is it more than a challenge?. Surg Endosc.

[18] Cugura JF, Janković J, Kulis T, Kirac I, Beslin MB (2008). Single-incision laparoscopic surgery (SILS) cholecystectomy: Where are we?. Acta Clin Croat.

[19] Prasad A (2010). Single-incision laparoscopic surgery. World J Gastroenterol.

